# Supplementary material for: Rosmarinic Acid-Grafted Dextran/Gelatin Hydrogel as a Wound Dressing with Improved Properties: Strong Tissue Adhesion, Antibacterial, Antioxidant and Anti-Inflammatory
Source: Molecules. 2023 May 11;28(10):4034. doi: 10.3390/molecules28104034 (PMC10223410; doi:10.3390/molecules28104034)
Supplement: Supplementary file 1 [file molecules-28-04034-s001.zip › molecules-2334511-supplementary.pdf]

Supplementary Materials

Rosmarinic acid grafted dextran/gelatin hydrogel as a wound dressing with improved properties: strong tissue adhesion, antibacterial, antioxidant and anti-inflammatory

Yi Yin[a], Qianqian Xu[a], Dongsheng Li[b*], Juanjuan Zhao[a*]

The number of pages：15

The number of figures：13

1. Experimental Section

1.1 Preparation and characterization of the oxidized dextran (ODex)

5 g dextran was stirred and dissolved in 50 mL deionized water at 37°C. The 15% $NaIO_4$ solution was prepared by dissolving 9.9 g $NaIO_4$ in 66 mL deionized water. Then the $NaIO_4$ solution was dropped into the dextran solution in the ice bath under the stirring of the magnet to avoid local overheating in dark condition. The reaction was allowed to proceed at 25°C with stirring for 4 h. After that, the reaction was terminated by adding 1 mL diethylene glycol and stirring for 15 min. The solution was dialyzed against distilled water for 48 h with several changes of water till the dialyzate was periodate-free. The dialyzate was then freeze dried.

The oxidation degree of ODex was determined by hydroxylamine hydrochloride titration. The structure was characterized by FT-IR, ^{1}H-NMR and UV-vis.

1.2 Preparation and characterization of amino-gelatin(AG)

5 g gelatin was stirred and dissloved in 200 mL NaH_2PO_4 (0.1 moL/L, pH 5.0) buffer solution at 37°C. ED·HCl was added into the clear solution. Then 2.3 g EDC was sequentially added. The reaction mixture was stirring at 37°C for 6 h. The modified gelatin was collected by dialysising and freeze drying.

The amino group content in the modified gelatin was determined by ninhydrin colorimetry. And the structure was characterized by FT-IR, ^{1}H-NMR and DSC.

1.3 Preparation of AG carrying Rosmarinic acid (AG−RA)

Firstly, RA was dissolved in ethanol and activated by adding EDC and NHS. The reaction was stirred in an ice bath for 1 h. Then the mixture was dropped into AG solution. The resultant mixture was keep stirring for 12 h at room temperature, followed by dialysis and freeze-drying.

The content of RA was determined by standard curve method. The structure of AG−RA was characterized by FT-IR, ^{1}H-NMR, UV-vis and DSC.

1.4 ^{1}H-NMR Spectrum Test

The spectra of ODex, AG, AG−RA were performed using a AVANCE-600 NMR instrument with deuter oxide serving as the solvent and internal standard.

1.5 FT-IR Spectrum Test

The ODex, AG, AG−RA and dried adhesive hydrogel were compressed into KBr pallet for spectroscopy measurement in the range of 4000-400 cm^{-1} by employing a Nicolet 6700 FT-IR spectrometer (Thermo Scientific Instrument).

1.6 UV-vis Spectrum Test

The spectrum of ODex, AG, AG−RA aqueous solution was recorded using a spectrophotometer in the range of 200-700 nm.

1.7 DSC measurements

Differential scanning calorimetry analysis on AG and AG−RA was performed using a 3 STARe System differential scanning calorimeter. About 5 mg of the each samples were weighed and covered into the alumina crucible, and the DSC curves were tested. The blank crucible was set as the reference. The heating rate was set as 10°C · min^{-1}, and the temperature range was 25~250 °C.

1.8 Determination of oxidation degree of ODex and amino content of AG

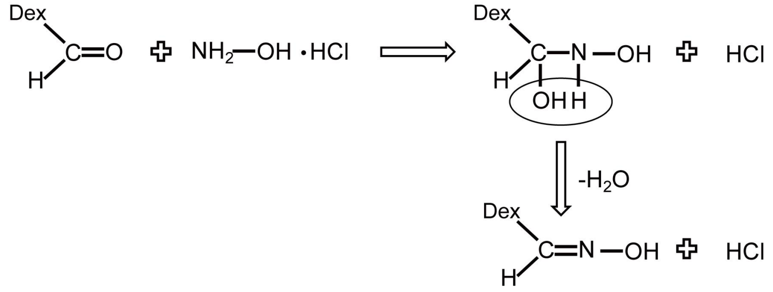

Figure. S1 Determination of oxidation degree of ODex by hydroxylamine hydrochloride method.

The oxidized degree (OD) of ODex was determined with a quantitative reaction between hydroxylamine hydrochloride and aldehyde groups, while producing a Schiff base and releasing hydrochloric acid (HCl). Through a titration method to calculate the amount of HCl, the concentration of aldehyde groups can be calculated. ODex sample (0.2 g) was mixed with 8 mL of hydroxylamine hydrochloride solution. A blank test was performed with 8 mL of hydroxylamine hydrochloride solution without ODex. The OD of ODex was calculated by:

$$\mathrm{OD} = \frac{162 \times C \times (V_0 - V_1)}{W} \times \frac{1}{2} \times 100\%$$

where C is the concentration of HCl solution (mol L^{-1}), V_0 is the volume of HCl used for the blank group (L), V_1 is the volume of HCl used for the sample (L), 162 is

the molecular weight of Dextran (g/mol), and W is the weight of the oxidation product added in the hydroxylamine hydrochloride solution (g).

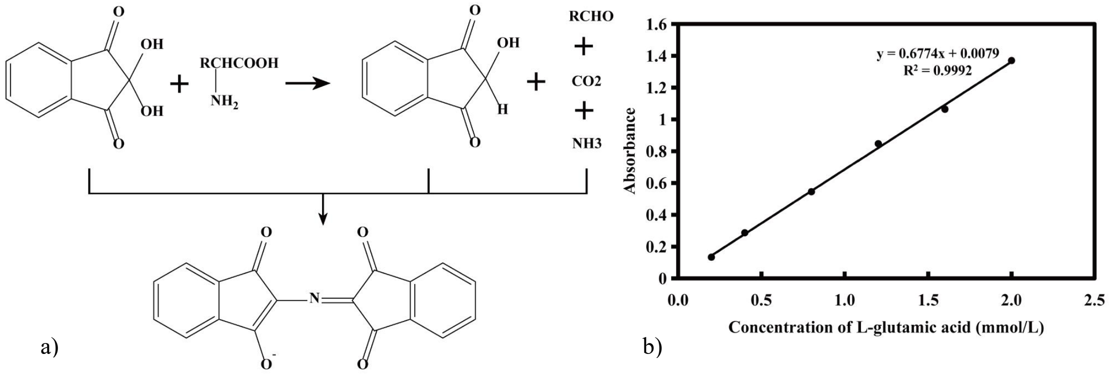

Figure. S2 Schematic diagram of color reaction between ninhydrin and amino acid (a); Standard curve of L-glutamic acid (b)

Ninhydrin method was performed to determine the amino content of gelatin and AG. The reaction of ninhydrin and α-NH_2 to produce a blue-purple substance. The reaction mixture has an absorption peak at 570 nm and the amino content is directly related to the absorbance. To determine the absorbance values of difffferent amino contents, a standard curve is constructed from the known concentrations of l-glutamate (Fig. S2). Then, the absorbance value of AG reacting with ninhydrin solution was measured and the amino content was determined.

1.9 Determination of content of RA in AG−RA

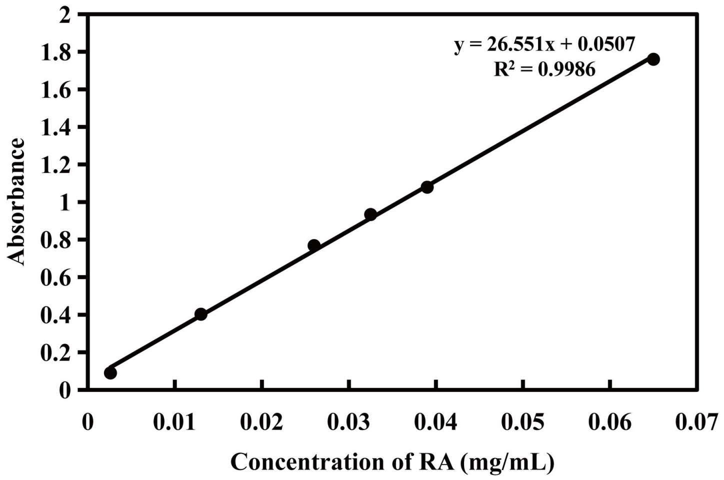

Figure. S3 Standard curve of RA

The content of RA in AG−RA was determined by RA standard curve method. RA has an absorption peak at 325 nm, and the RA content was determined according to the absorbance value at 325 nm.

1.10 Preparation of ODex-AG−RA hydrogels

Firstly, ODex, AG, AG−RA were dissloved in PBS (pH 7.4) with concentration of 0.15 g/mL, 0.30 g/mL, 0.30 g/mL, respectively. The hydrogels were prepared by mixing this two solutions homogenously in the mold using a vortex oscillator. The volume ratio of ODex solution and AG−RA solution was 0.5:1, 1:1, 2:1, respectively. After full stirring, the mixture formed hydrogels with a few seconds. For the convenience of subsequent statements, we called each of the three groups of hydrogels ODex−AG−RA−0.5, ODex−AG−RA−1, ODex−AG−RA−2 respectively. The hydrogel based on ODex and AG at the ratio of 1:1 was also prepared as the control sample and abbreviated as ODex−AG−1. The ODex−AG−1 and ODex−AG−RA−1 hydrogels were crushed to a powder after freeze-dried. The functional groups of hydrogels were identified in the region of 4000-400 cm^{-1} by FT-IR spectroscopy.

1.11 Gelation time test

The gelation time was determined by vial inversion method. ODex, AG or AG−RA solutions were added into the sample bottle in different proportions and mixed evenly through a vortex oscillator. Start timing after mixing evenly, and the gelling time was noted as the time when the inverted sample no longer flowed. All the gelation experiments were carried out at room temperature.

1.12 Morphology analysis

The four groups of hydrogel samples were freeze-dried and the dry hydrogels were broken with liquid nitrogen. Then the sections were sprayed with a gold layer. The micro morphology of the cross section was observed by scanning electron microscope (EVO LS15, ZEISS). Image J software was employed to measure the pore diameters of hydrogel samples.

1.13 Swelling test

The lyophilized hydrogels were weighed as Wd, and the hydrogels were

immersed in the PBS solution (pH7.4) at 37°C. Removed excess water from hyrdogels surface and weighed the swollen hydrogel samples at regular intervals. The weight at different time points were recorded as Ws (g). The swelling rate of hydrogel was calculated by the following formula.

$$\mathrm{SR}(\%)=\frac{\left(W_{\mathrm{s}}-W_{\mathrm{d}}\right)}{W_{\mathrm{d}}}\times 100$$

Where the Ws is weight of swollen hydrogels and Wd represent the weight of dry hydrogels.

1.14 In vitro degradation test

The prepared hydrogels were accurately weighed and immersed in the PBS solution for cultivation in an incubator at 37°C. Freeze dried and weighed the hydrogels at regular intervals (on days 1, 3, 5, 7, 14, 21 and 28). The weight remaining rate (%) of the hydrogel was determined by the following equation.

$$\mathrm{WR}(\%)=\frac{W_{\mathrm{t}}}{W_0}\times 100$$

Where the W_t was dry weight of the remaining hydrogel at different time points, and W_0 was dry weight of the initial hydrogel.

1.15 Rheological test

Rheological measurements were performed on a TA rheometer (DHR-1) under a oscillation model. A cone-plate geometry with a diameter of 40 mm was used. 800 μL precursor solutions of hydrogels were added between the parallel plates. For time sweep tests, the storage moduli G' and loss moduli G" of the hydrogels were monitored as a function of time at a frequency of 1 rad/s and a shear strain of 1% at a constant temperature of 25°C.

1.16 Adhesion strength test

The adhesion property of hydrogels was tested on porcine skin surfaces through a lap shear test. Briefly, 50 μL of precursor solution of hydrogels was applied onto the surfaces of the two porcine skins (1cm×3cm). The adhesive area was kept as 1cm×1cm. Overlap this area for 1 hour to ensure complete bonding. The hydrogel was cross-linked between the gelatin sheet and porcine tissue. The pigskin was fixed on a

universal testing machine for tensile test at a speed of 1 mm/min until it was separated. All these tests were employed 3 times.

1.17 In vitro antioxidant activity of hydrogels

In vitro antioxidant activity of the hydrogels was analyzed by DPPH radical scavenging, superoxide anion radical scavenging and total reducing power.

(1) DPPH radical scavenging

Briefly, 0.8 mL of freshly prepared DPPH ethanol solution (0.1 mM) was thoroughly mixed with 200 uL of the four groups of hydrogels, respectively. The reaction was kept in a dark place for 30 min at room temperature. The absorbance of the supernatant was measured at 517 nm, and VC solution (3 mg/mL) was used as the positive control. The scavenging of DPPH was calculated by the following formula:

$$\text{DPPH scavenging (\%)} = \left(1 - \frac{A_1 - A_2}{A_0}\right) \times 100$$

Where A_0, A_1 were the absorption of the blank group (DPPH + PBS) and the absorption of the sample groups (DPPH + hydrogel/VC), respectively. A_2 was the absorption of the mixture of ethanol and hydrogels/VC.

(2) Superoxide anion radical scavenging

1 mL hydrogel was mixed with 1 mL NBT (156 μM), 1 mL NADH (200 μM) and 1 mL PMS (60 μM). The mixture was incubated for 5 min at room temperature. Next, absorbance at 560 nm was subsequently measured using a microplate reader. All the above reserve solutions were prepared with Tris-HCl buffer (16 mmoL/L, pH 8.0), and VC solution (3 mg/mL) was used as a positive control. The scavenging of superoxide anion radical was calculated by the following formula:

$$\text{superoxide anion radical scavenging (\%)} = \left(1 - \frac{A_1 - A_2}{A_0}\right) \times 100$$

Where A_1, A_2 and A_0 represent the absorbance of sample reaction solution, reserve solution (hydrogel+Tris-HCl buffer) and PBS control group, respectively.

(3) Total reducing power

1 mL 0.2 mol/L phosphate buffer (pH 6.6) and 1 mL of 1% (w/v) potassium ferricyanide solution were added to 0.2 mL of the hydrogel sample. The mixture was

stirred and reacted in a water bath at 50℃ for 20 min, followed by rapid cooling. Then 1 mL of 10% trichloroacetic acid solution was added, and the mixture was shaked evenly and incubated for 10 min. 1 mL of the supernatant was fixed with 1 mL of deionized water and 0.2 mL of 0.1% ferric chloride solution. Finally, the mixture absorbance was measured at 700 nm after evenly mixing. The absorbance value is directly used to evaluate the strength of reducibility.

1.18 Antibacterial activity of hydrogels

(1) *Escherichia coli* (*E. coli*) and *Staphylococcus aureus* (*S. aureus*) were used to test the antibacterial activity of hydrogels. 100 μL hydrogel was mixed with 1 mL of bacterial suspension (10^6 CFU mL^{-1}) in a 24-well plate. 1 mL of 10^6 CFU mL^{-1} bacterial suspension without hydrogel was used as a control. The well plates were incubated for 24 h in a 37℃ incubator at 150 rpm. After 100 times dilution of the mixture solution, 100 μL diluted solution was spread on an agar plate. After 24 h culture, the colony forming unit (CFU) was calculated on the petri dish to evaluate the in vivo antibacterial property.

$$\text{Bacterial survival rate (\%)} = Ne/Nc \times 100\%$$

Where the Ne was the number of bacteria in hydrogel group, and Nc was the number of bacteria in control group.

(2)100 uL of hydrogel samples and 6 mL 10^6 CFU mL^{-1} of bacterial suspension were mixed in a 50 mL conical flask and cultured for 24 h. After coculture, the bacterial suspensions were washed twice with PBS and resuspended in 1.0 mL of PBS (pH 7.4). Subsequently, the bacteria were stained with the FDA (10 ug/mL) and PI (1 mg/mL) for 30min, and then washed 3 times with PBS. Finally, 10 μL bacterial suspensions was dropped onto the slide, the fluorescence images were captured on an Olympus optical microscope (Japan).

(3) The morphology of *E. coli* and *S. aureus* was further observed using SEM. Generally, the hydrogel was co-cultured with bacterial solution according to the method in 1.18 (1), and the hydrogel was taken out and washed with PBS. As a control, the same amount of bacterial solution was cultured in PBS and centrifuged. And then fixed hydrogels and bacterial with glutaraldehyde (2.5%) overnight at 4°C.

The fixed bacteria were then dehydrated by sequential treatment with 30%, 50%, 70%, 80%, 95%, and 100% ethanol (8 min for each gradient). A droplet of the bacteria suspension from the control groups was added on a piece of Polylysine slide (1 cm^2) and then dried in the oven. Finally, the morphology of bacterial in hydrogel samples and slide were imaged using a SEM after drying.

(4) Hydrogels of various proportions were prepared in each well of the 24-well plate. 75% ethanol was added under ultraviolet irradiation for sterilization for 2 h, after the gel was stabilized. And then the hydrogels were soaked in PBS for 2 h. 1 mL 10^6 CFU mL^{-1} bacterial suspension was added to each well. The growth state of the biofilm was observed after incubation at 37°C for 24 h, 48 h and 72 h. The liquid medium was abandoned at each time point, and the unattached bacteria were washed off with PBS. And then the biofilm was stained with 0.2% crystal violet solution at room temperature for 30 min. After staining, the excess dye was cleaned with 1 mL PBS. Finally, the material was dried and dissolved with 95% ethanol for 15 min followed with dilution. The absorbance was measured at 590 nm.

1.19 Hemolytic test of hydrogels

Blood of Kunming mice was collected in EP tubes infiltrated with heparin sodium. 1mL of blood was centrifuged to separate red blood cells from serum and washed the blood cells with sterile PBS (pH 7.4) three times (1000 rpm, 10 min). Red blood cell suspension was prepared by adding red blood cells to 30 mL of PBS.

200 μL of each hydrogel samples was placed at 24-well plate after UV irradiation. 1 mL of the blood cell suspension was added to the hydrogel of the 24-well plate and incubated at 37°C for 1 h. TritonX-100 was used as positive control while PBS buffer was used as the negative control. Then, the samples were centrifuged at 1000 rpm for 10 min, and the OD values of the supernatant (100 μL) were measured at 540 nm with a microplate reader. The hemolysis rate (HR) was calculated using the following formula :

$$HR(\%)= (OD_s-OD_-)/(OD_+-OD_-)\times 100\% \qquad (4)$$

Where OD_s was the absorbance value for hydrogel samples. OD_+ was the absorbance value for the TritonX-100 positive control and OD_- was the absorbance value for PBS negative control.

1.20 In vitro cytotoxicity

Leaching solution method was used to determine the cytotoxicity of hydrogels. 100 μL of the hydrogels were completely immersed in 1mL Dulbecco's modified Eagle's medium(DMED) , and the leaching solution was obtained at 37°C for 24 hours. The complete culture medium was prepared by adding 10% fetal bovine serum (FBS) and 1% Penicillus-streptomycin. A series of sterilized ODex−AG−1, ODex−AG−RA−0.5, ODex−AG−RA−1, ODex−AG−RA−2 hydrogels leaching solution with concentrations of 0.625%, 1.25%, 2.5%, 5% (v/v) were prepared.

L929 cells (8×10^4/mL) of logarithmic phase were inoculated on 96 well culture plate and incubated at 37°C for 24 h in a humidified atmosphere with 5% CO_2. The culture medium was then replaced by the leaching solution, and the cells were incubated for an additional 24 h. Cells seeded by the complete growth medium served as the control group. After being co-incubated for 24 h, the culture medium was removed and CCK-8 solution (10%) was added to each well. The microplate reader (Synergy H1, Bio Tek, USA) was used to measure the light absorption value at the wavelength of 450 nm after two hours.

For live/dead staining, cells were seeded in 24-well plates and the treated processing was the same as above. The supernatant was removed and 250 μL of Calcein-AM/PI staining solution was added to each well. After incubating for 30 min, the fluorescent images of the cells were observed using a fluorescence microscope (DMI4000, Olympus, Japan).

1.21 In vivo wound closure evaluation

Male SD mice weighting about 250 g and 6 week age were used for studies. All rats had free access to sterile water and standard chow to acclimatize for one week before surgery. After inducing anesthetized by intraperitoneal injection of 10% chloral hydrate at a dose of 1 mL/300 g, the dorsal region of mice were shaved. Subsequently, four round full-thickness skin wounds of approximately 13 mm diameter were made

by surgical scissors on both sides of the dorsal spine of the rats. The skin wounds covered with the Tegaderm^{TM} hydrogel were used as control group, and the hydrogel groups were added with 50μL ODex−AG−1 hydrogel or 50 μL ODex−AG−RA−1 hydrogel. The wounds without treatment were used as the blank control group. After the treatments, the wounds were protected with Tegaderm^{TM} film and gauze. The rats were housed in separate cages and the wound recovery was recorded on 3rd, 7th, 14th and 21th days after operation by digital camera. The area of the wound was measured by Image J and the wound healing rate was calculated using the following formula:

$$\text{Wound healing rate (\%)} = (A_0 - A_n)/A_0 \times 100\%$$

Where A_0 and A_n were the initial wound area and the wound area at different time points, respectively.

1.22 Histology and immunohistochemistry

The skin closure and regeneration were evaluated by histopathologic examination. The animals were sacrificed at 3rd, 7th, 14th and 21th days after operation, and the tissue samples were collected and fixed in paraformaldehyde. After dehydration, the tissue samples were embedded in paraffin and cut into 5-μm-thick sections. Then hematoxylin and eosin (H&E) staining and Masson's trichrome staining were performed to observe inflammation reaction, tissue regeneration and collagen deposition under microscope. The regenerated skins from the wound site were also excised at 7th and 14th day for immunofluorescence and immunohistochemistry staining. The fixed sections were stained with TNF-α and CD163 to evaluate inflammatory cell infiltration. Besides, the tissue sections on day 14 were stained with immunofluorescence for CD31 for blood vessels regeneration.

1.23 Determination of antioxidant indexes in vivo

The antioxidant properties of hydrogels were evaluated by determining the content of MDA and H_2O_2 according to the steps of the kit.

2. Results

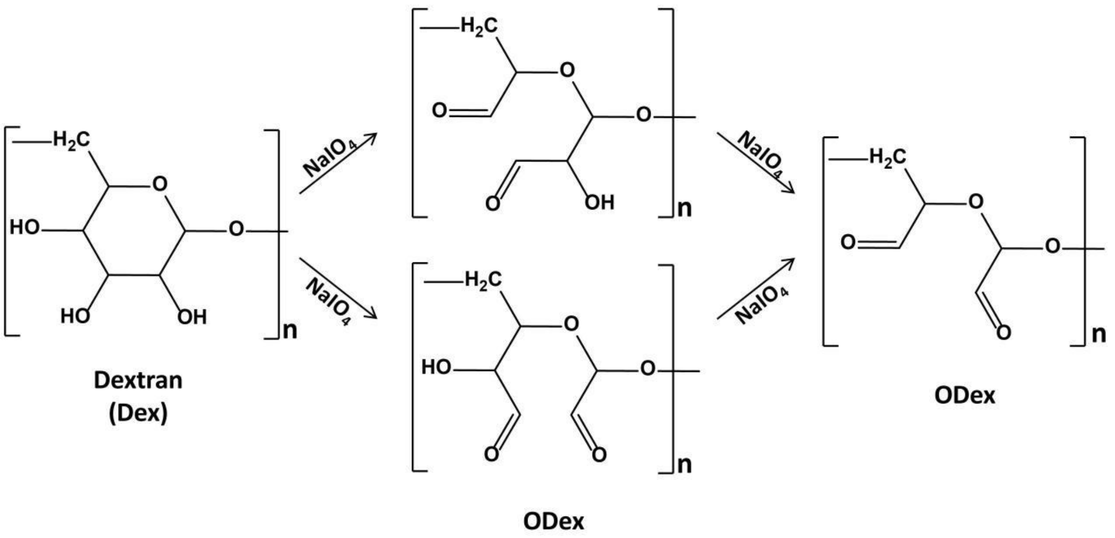

Figure S4. Synthesis of ODex

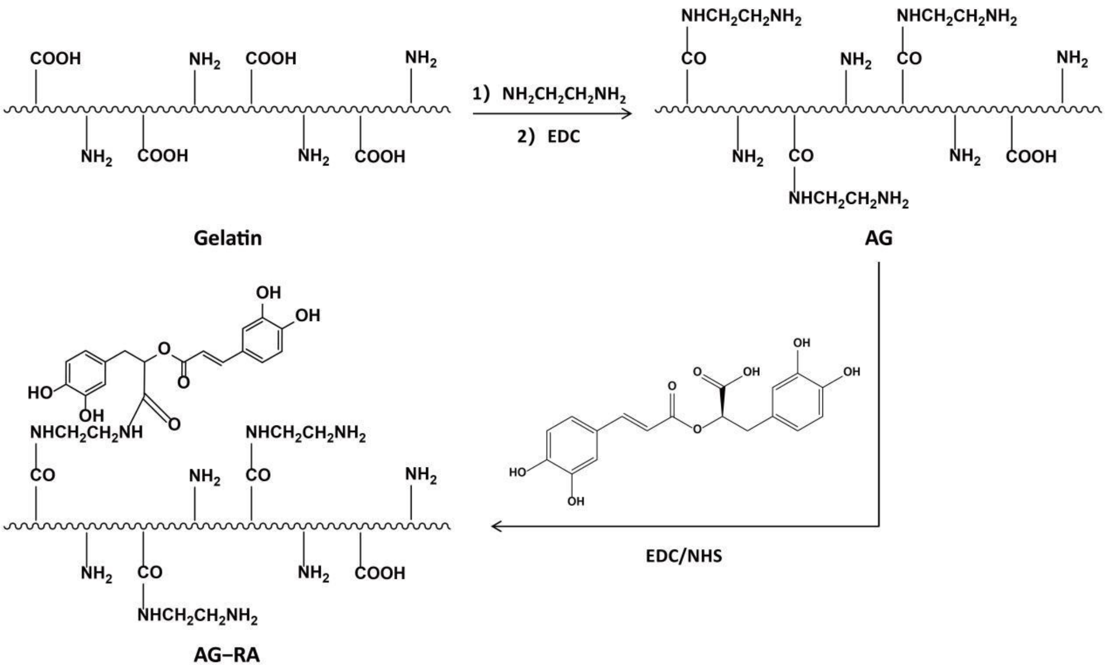

Figure. S5. Synthesis of AG and AG−RA

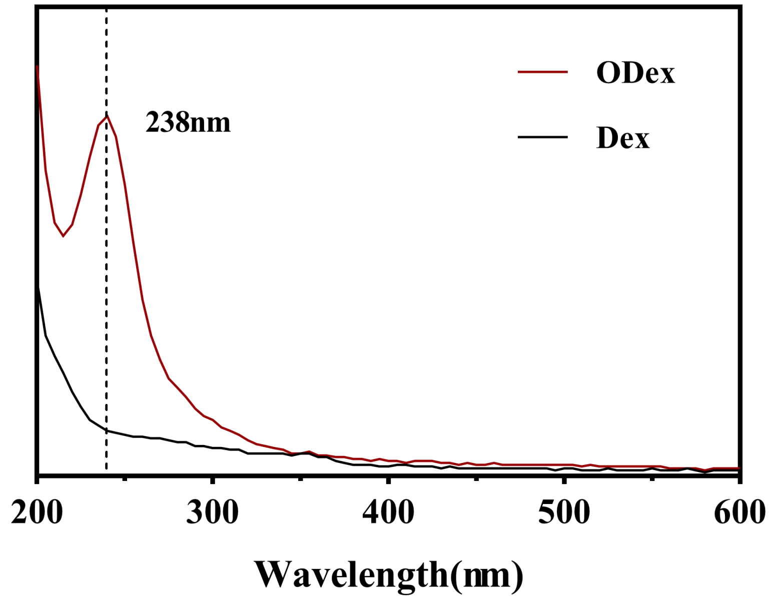

Figure. S6. UV-vis absorption spectra of Dex and ODex.

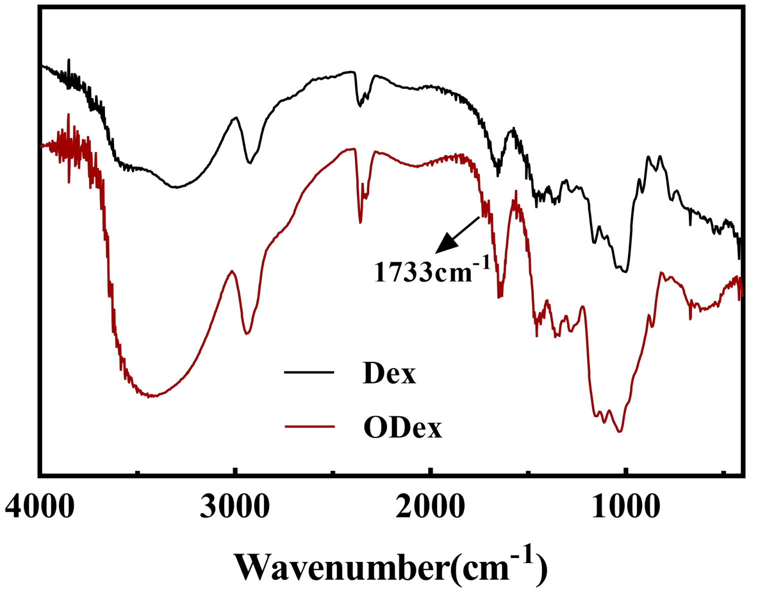

Figure. S7. FT-IR absorption spectra of Dex and ODex.

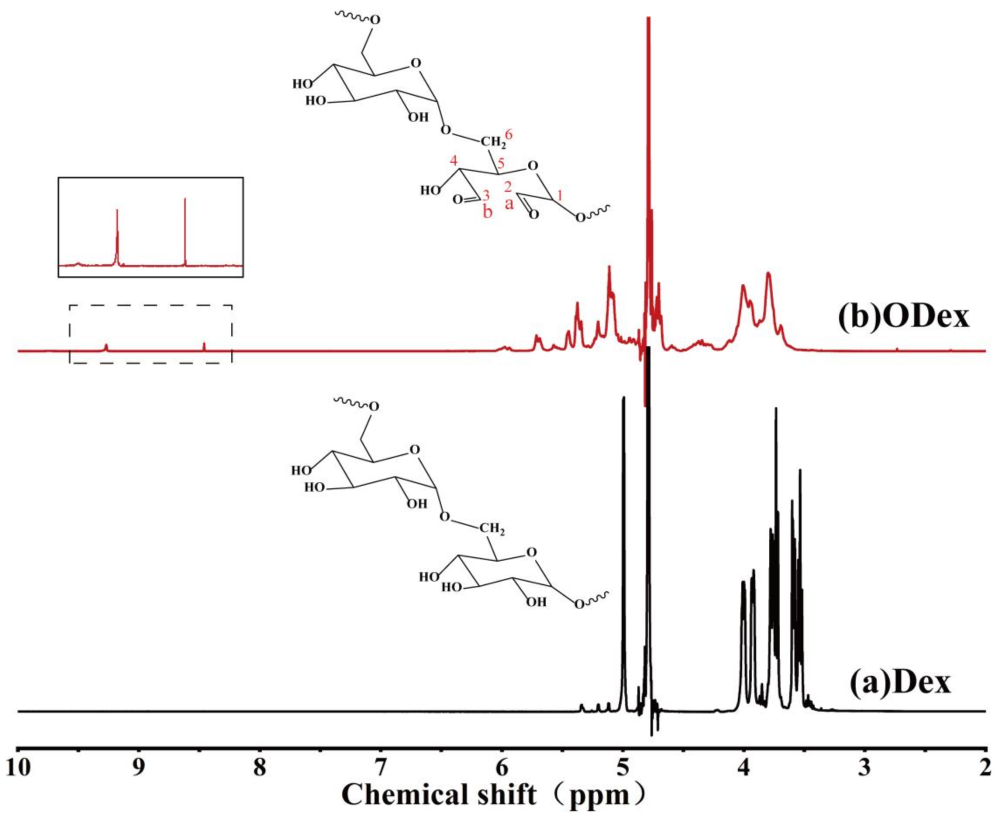

Figure. S8. ^{1}H-NMR absorption spectra of Dex and ODex.

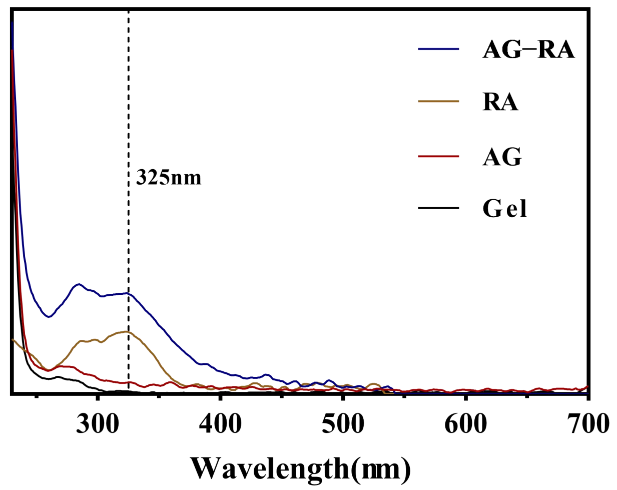

Figure. S9 UV-vis absorption spectra of Gelatin, AG and AG−RA

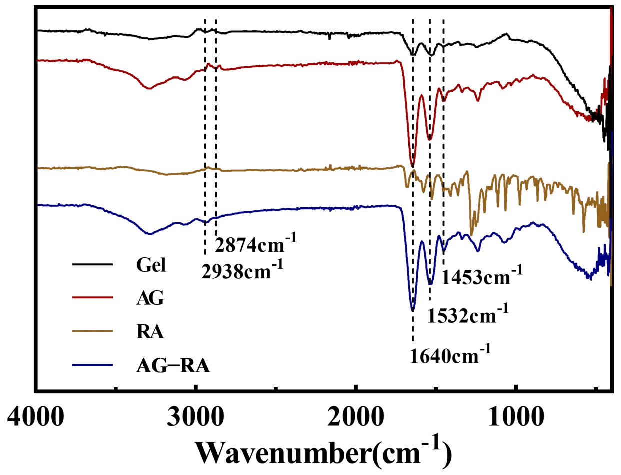

Figure. S10 FT-IR absorption spectra of Gelatin, AG and AG−RA

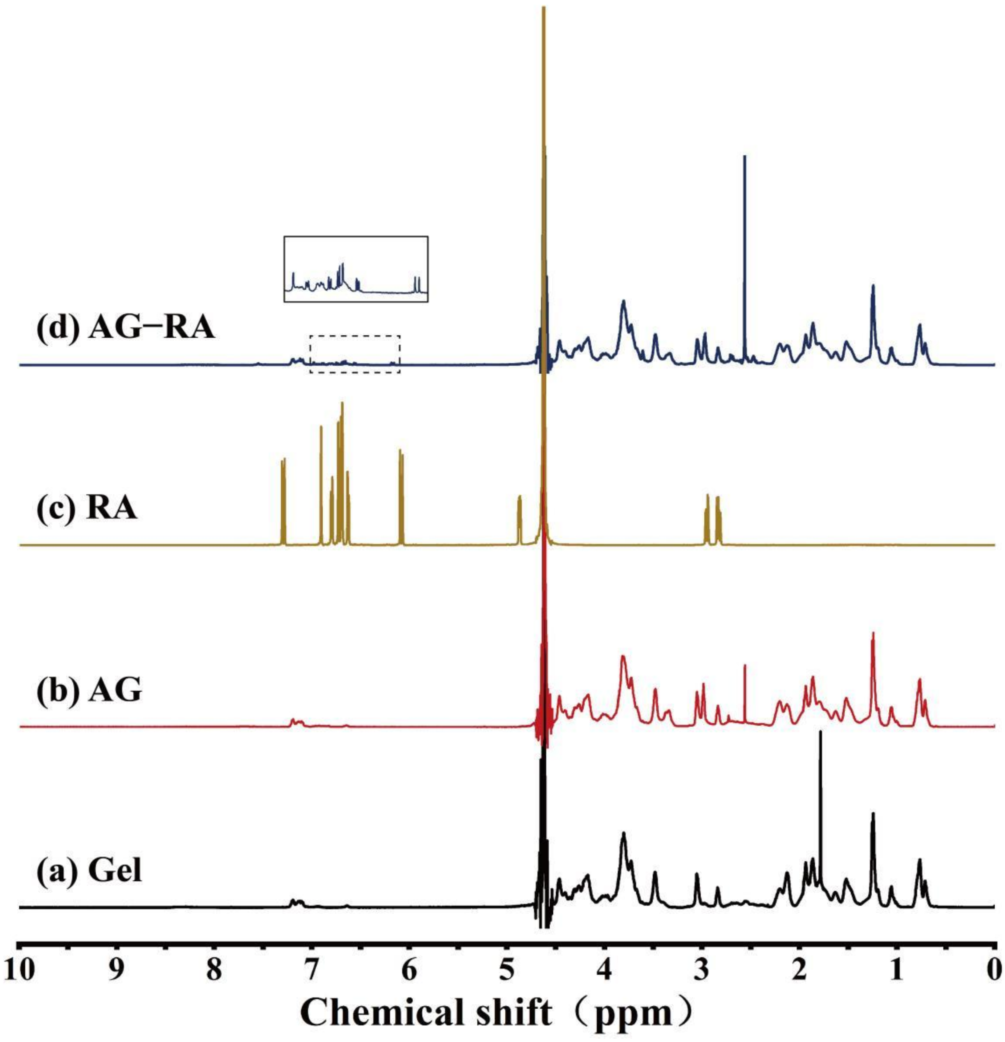

Figure. S11 ^{1}H-NMR absorption spectra of Gelatin, AG and AG−RA

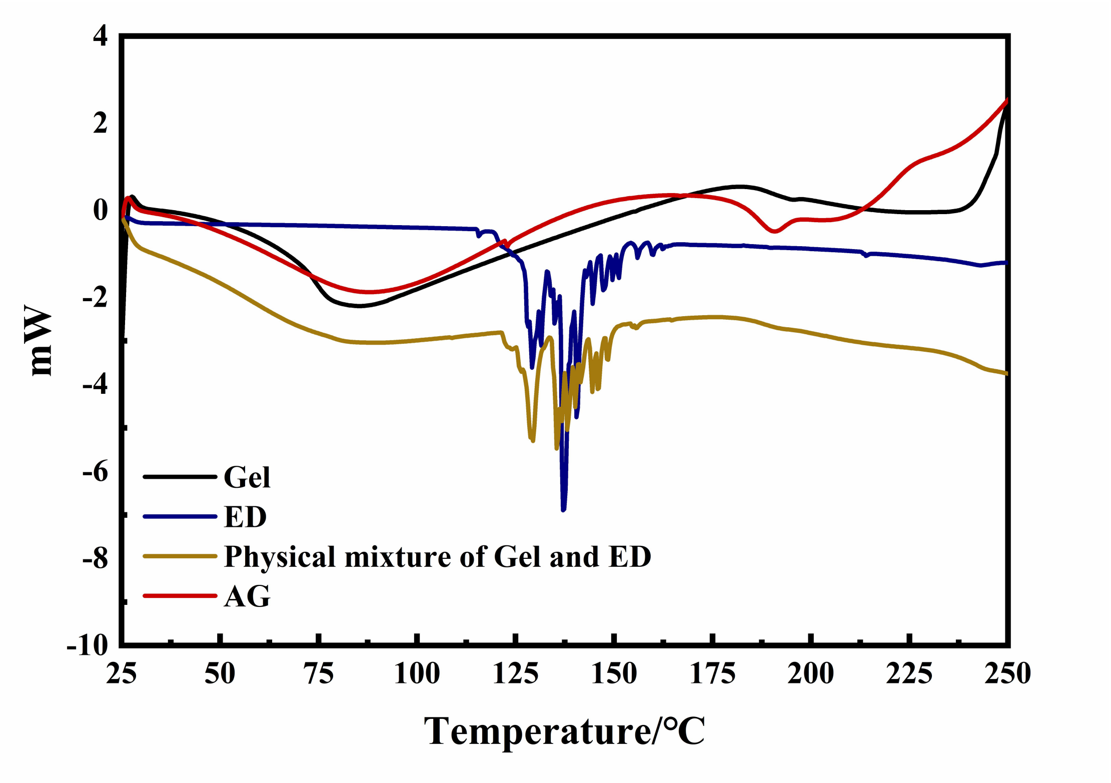

Figure. S12 DSC curves of gelatin and AG (Gelatin before and after amination)

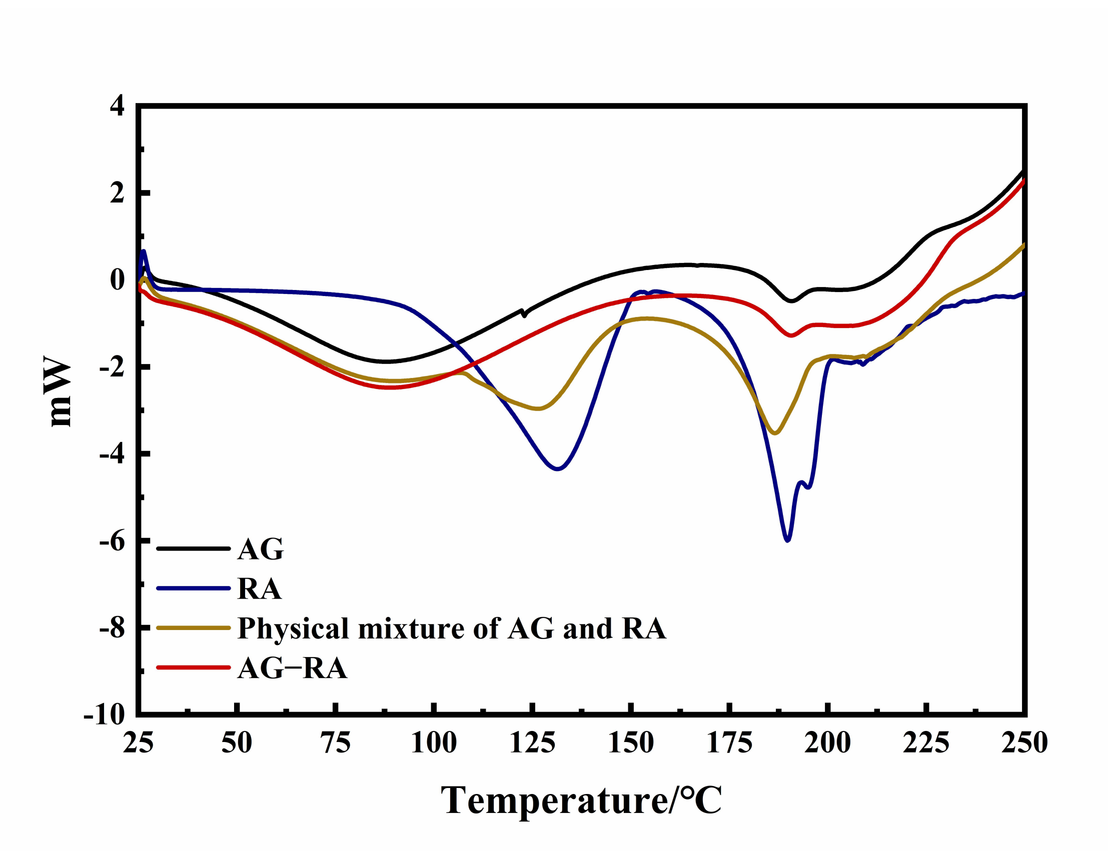

Figure. S13 DSC curves of AG and AG−RA (AG before and after RA grafting)
